# Assessment of Retinal Capillary Dropout after Transcatheter Aortic Valve Implantation by Optical Coherence Tomography Angiography

**DOI:** 10.3390/diagnostics11122399

**Published:** 2021-12-20

**Authors:** Jeanne Martine Gunzinger, Burbuqe Ibrahimi, Joel Baur, Maximilian Robert Justus Wiest, Marco Piccirelli, Athina Pangalu, Dominik Straumann, Fabian Nietlispach, Igal Moarof, Sandrine Anne Zweifel

**Affiliations:** 1 Department of Ophthalmology, University Hospital of Zurich, University of Zurich, 8091 Zurich, Switzerland; joel.baur@vista.ch (J.B.); maximilian.wiest@usz.ch (M.R.J.W.); sandrine.zweifel@usz.ch (S.A.Z.); 2 Department of Cardiology, University Hospital of Zurich, University of Zurich, 8091 Zurich, Switzerland; burbuqe.ibrahimi@ksb.ch (B.I.); fabian.nietlispach@hirslanden.ch (F.N.); 3 Department of Information Technology and Electrical Engineering, ETH Zurich, 8092 Zurich, Switzerland; piccirelli@biomed.ee.ethz.ch; 4 Department of Neuroradiology, University Hospital of Zurich, University of Zurich, 8091 Zurich, Switzerland; athina.pangalu@usz.ch; 5 Department of Neurology, University Hospital of Zurich, University of Zurich, 8091 Zurich, Switzerland; dominik.straumann@usz.ch; 6 Heartcenter im Park, Hirslanden Clinic Im Park, 8027 Zurich, Switzerland; 7 Department of Cardiology, Kantonsspital Baden, 5404 Baden, Switzerland; igal.moarof@ksb.ch

**Keywords:** optical coherence tomography angiography, retinal emboli, transcatheter aortic valve implantation, OCTA, TAVI

## Abstract

Transcatheter aortic valve implantation (TAVI) is an alternative to open heart surgery in the treatment of symptomatic aortic valve stenosis, which is often the treatment of choice in elderly and frail patients. It carries a risk of embolic complications in the whole cerebral vascular bed, which includes the retinal vasculature. The main objective was the evaluation of retinal emboli visible on optical coherence tomography angiography (OCTA) following TAVI. This is a prospective, single center, observational study enrolling consecutive patients over two years. Patients were assessed pre- and post-TAVI. Twenty-eight patients were included in the final analysis, 82.1% were male, median age was 79.5 (range 52–88), median BCVA was 82.5 letters (range 75–93). Eight patients (28.6%) presented new capillary dropout lesions in their post-TAVI OCTA scans. There was no statistically significant change in BCVA. Quantitative analysis of macular or peripapillary OCTA parameters did not show any statistically significant difference in pre- and post-intervention. In conclusion, capillary dropout lesions could frequently be found in patients after TAVI. Quantitative measurements of macular and peripapillary flow remained stable, possibly indicating effective ocular blood flow regulation within the range of left ventricular ejection fraction in our cohort.

## 1. Introduction

Transcatheter aortic valve implantation (TAVI) is an alternative to open heart surgery in the treatment of symptomatic aortic valve stenosis [1]. A crimped bioprosthetic valve is delivered to the native aortic valve annulus on a stent, where the valve stent is expanded, pushing the native aortic valve aside. The procedure can be performed in local anesthesia. TAVI is the treatment of choice for the elderly and often multimorbid patient. However, perioperative risk for embolic complication (mostly collagenous tissue or thrombotic material) [1] is high: postoperative stroke affects 3.3% (1.4% to 9%) of patients [2,3,4], while clinically silent ischemic lesions in the brain appear in 70–80% of patients [5,6,7].

Emboli to the cerebral vasculature are spread in all beds of the cerebral vasculature (carotid and vertebral vasculature), and therefore most likely also affect the retinal vessels (e.g., microemboli, central retinal artery occlusion, branch retinal artery occlusion or even septic emboli) [8,9,10]. Retinal artery occlusion is associated with a significantly higher incidence of stroke [11]. Thereby, retinal (micro) emboli may be a surrogate marker for the total embolic burden to the cerebral vasculature.

Improvements in optical coherence tomography angiography (OCTA) over the last years have allowed for wide-field high-resolution visualization of the retinal and choroidal perfusion in minimal acquisition time [12,13,14,15]. This is said to also make the imaging more feasible for the elderly and patients in moderately reduced general condition. The main objective of the here presented prospective, single center, observational study was the evaluation of retinal microemboli visible on OCTA following TAVI. Secondary objectives were general retinal perfusion changes and the correlation of the OCTA findings with cerebral ischemic lesions on MRI.

## 2. Materials and Methods

This is a prospective, single center, observational study, approved by the institutional review board approved this study (Cantonal Ethics Committee, Canton of Zurich, Switzerland, BASEC-No. 2017-00641) and carried out in accordance with principles of the Declaration of Helsinki (DoH) and the Essentials of Good Epidemiological Practice issued by Public Health Switzerland (EGEP). A written informed consent for the processing of personal data was obtained from each patient. All inpatients receiving transcatheter aortic valve implantation (TAVI) between June 2017 and November 2019 at the Heart Center at the University Hospital Zurich (USZ) with a minimal best-corrected visual acuity (BCVA) of 50 letters (according to the Early Treatment of Diabetic Retinopathy Study, ETDRS) in both eyes and no contraindication for MRI were eligible for inclusion. Cardiologists from the Heart Center recruited patients, which were then screened for eligibility at the Department of Ophthalmology at the USZ. Patients had one screening visit, one baseline exam before TAVI, and one follow up exam within seven days after TAVI.

For the assessment of the primary endpoint (i.e., microemboli on OCTA), we used swept source OCTA wide field recording (Plex Elite 9000, Carl Zeiss Meditec AG, Jena, Germany). Study protocol included macular 3 × 3 mm and 6 × 6 mm, disc 6 × 6 mm and temporal 6 × 6 mm, posterior pole 15 × 9 mm and panorama reconstruction using five 12 × 12 mm scans. Images were analyzed qualitatively for capillary dropout lesions as representatives for areas affected by microembolies. All images were assessed for new capillary dropouts (binary yes or no) after TAVI.

The secondary endpoint correlation with cerebral ischemic lesions was evaluated by acquiring magnetic resonance imaging (MRI) of the brain (diffusion weight, flair, and T2 sequences) performed and analyzed at the Department for Neuroradiology at the USZ.

The secondary endpoint general retinal perfusion changes was analyzed by quantitative measurements of macular 3 × 3 mm, macular 6 × 6 mm, and disc 6 × 6 mm superficial (SCP), and deep capillary plexus (DCP) scans only, as the larger scans do not have sufficient resolution for quantitative analysis. All scans were assessed for vessel density and vessel length density. Additionally, macular 3 × 3 mm scans were assessed for foveal avascular zone (FAZ). Macular scans were processed using algorithms provided by Carl Zeiss Meditec AG. Peripapillary scans were processed using FIJI (“Fiji Is Just ImageJ”, open source image processing package, https://imagej.net/, accessed on 26 September 2021, Version 2019), with an inner and outer ring around the disc measuring 1 mm each (ImageJ macros designed at USZ) [16].

A post hoc analysis was added using heat maps on posterior pole 9 × 15 mm scans, a data visualization technique using color hues to better indicate flow intensities (this was not included in the original protocol because the algorithm, became available later during the study period). The implemented algorithm was provided by Carl Zeiss Meditec AG.

Other study variables such as demographics and medical/ophthalmological history were assessed by questionnaire or extracted from patients file at the screening visit. Main focus was on ophthalmologic history (age-related macular degeneration (AMD), cataract, glaucoma, retinopathies, and trauma), and history of arterial hypertension or diabetes. Details to the TAVI and postoperative complications were extracted from the patient’s medical file at the follow up exam.

Statistical analyses were performed as a self-controlled model, using SPSS Statistics (Version 26.0, IBM Corp, Armonk, New York, NY, USA). The primary comparison groups were the data from the baseline visit to the data from the follow-up visit. To calculate the summary statistics of continuous variables, measurements of two eyes from the same patient were averaged (sample mean and median coincide with only two measurements [17]. To assess the effect of the intervention on the measurements of the eye, the change score, defined as the follow-up (or post-operative) value minus the baseline (or pre-operative) value, was created.

## 3. Results

**Figure 1:**
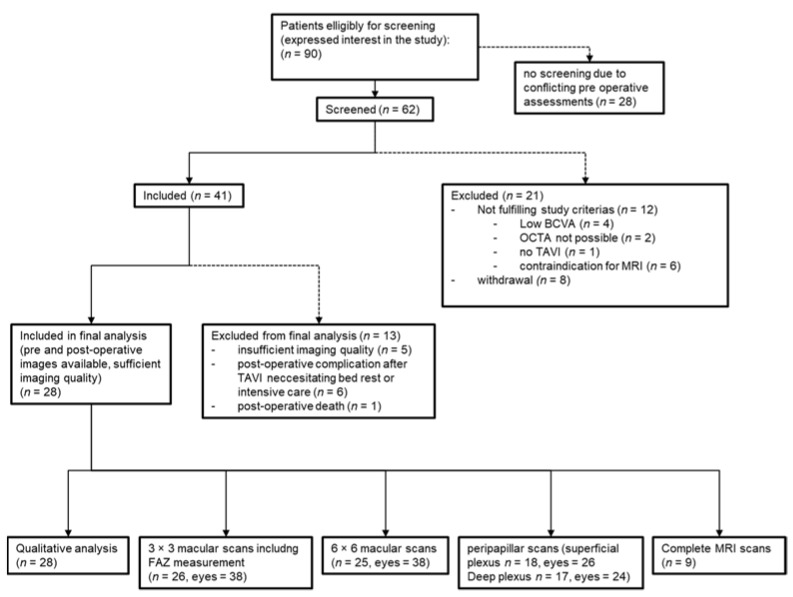
Flowchart of the patient enrollment process of the study cohort. BCVA = best corrected visual acuity. OCTA = optical coherence tomography angiography. TAVI = transcatheter aortic valve implantation. MRI = magnetic resonance imaging. FAZ = foveal avascular zone.

Ninety patients scheduled for TAVI expressed interest in participating in the study. Sixty-two patients were screened, whereof forty-one patients were eligible and included in the study. Additional eight patients were first included, but then withdrew from participation during the study to a later point. Out of the forty-one patients included, twenty-eight had good quality pre- and postoperative OCTA scans available for analysis in at least one eye. Thirteen (31.7%) were excluded from analysis, mainly due to missing postoperative scans due to TAVI related complication, necessitating intensive or intermediate care or poor quality of the images (Figure 1). Primary reason for poor image quality was high quota of motion artifact, making analysis unfeasible. Mean time between TAVI and OCTA follow up exam was four days (SD = 1.5 days).

### 3.1. Analysis of Demographics

Twenty-eight patients were included in the final analysis, 82.1% were male. Median age was 79.5 years (range 52–88), median BCVA at baseline was 82.5 letters (range 75–93). Eight patients were pseudophakic (both eyes), five had known dry age-related macular degeneration (mild or intermediate). A total of 85.7% suffered from arterial hypertension, 25.0% were diabetic, none of which presented clinically significant diabetic macular edema nor proliferative diabetic retinopathy; 7.1% had a history of stroke. Reason for TAVI was aortic stenosis in all patients, for two patients it was their second aortic valve implantation. Most patients had high grade severe aortic stenosis (53.6%), followed by low flow low gradient aortic stenosis (25.0%). Detailed baseline demographics are presented in Table 1.

### 3.2. Qualitative Analysis

**Figure 2:**
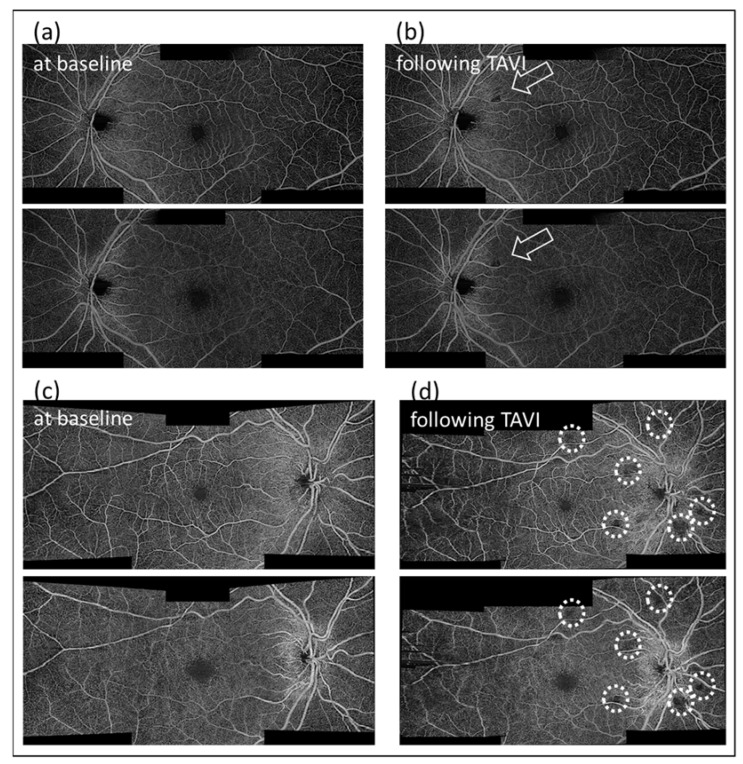
Examples of capillary dropout lesions on OCTA. (**a**) and (**c**) are images acquired at baseline of two exemplary cases (71- and 76-year-old patients, both male). These posterior pole images are reconstructed from three individual 6 × 6 mm scans—a macular, a temporal and a papillary scan of the superior and deep capillary plexus. (**b**) post-TAVI OCTA reconstructed image of the same subject as image (**a**), presenting a singular capillary dropout lesion (arrow). (**d**) Post-TAVI OCTA reconstructed image of the same subject as image (**c**), presenting multiple capillary dropout lesions (circles). Please also note the motion artifacts on the temporal (left) edge of the image (**d**), which present as horizontal lines/image shifts due to the image acquisition in horizontal line scans (in Plex Elite 9000). These motion artifacts were more prevalent in peripheral scans, most likely as it is more difficult to keep the gaze steady in non-central fixation. These motion artifacts are very recognizable and distinctive from the drop out lesions, which are roundish and do not show horizontal borders and are not in extension of the horizontal lines.

Eight patients (28.6%) presented new capillary dropout lesions in their post TAVI OCTA scans (Figure 2). All of them only showed capillary dropout lesions in only one eye, mostly a single area. The drop out lesions were always visible in the SCP and the DCP. Most of those lesions were either within or close to the vascular arcade or within three mm of the disc. Note that most of the more peripheral wide-field images acquired were of poorer quality and therefore not reliable for evaluation.

Nine patients (32.1%) had pre- and post-TAVI MRI. The other candidates either withdrew from undertaking MRI on the examination day or were medically unstable to do so after TAVI or had new contraindications for MRI (i.e., pacemaker implantation during TAVI). Eight (88.9% of patients with MRI or 28.6% of all patients) presented (silent) ischemic lesions, one had no ischemic lesion. Out of the eight patients presenting silent ischemic brain lesions, two had detectable capillary dropout lesions on OCTA, whereas the other six did not show any qualitative difference in their post-TAVI scans. Due to missing data in 67.9%, we waived calculating the correlations between MRI and OCTA findings.

### 3.3. Quantitative Analysis

**Figure 3:**
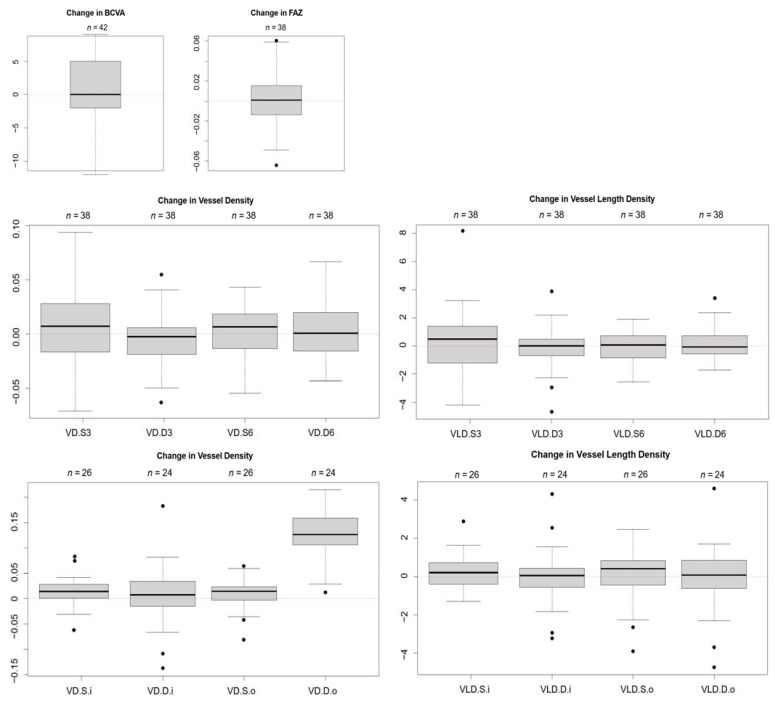
Quantitative analysis of OCTA: Change in measurements (pre- and post-TAVI) of vessel density and vessel length density of the superficial and deep plexus did not show a statistically significant difference, the values oscillate around zero. *n* gives the number of eyes included for the specific quantitative analysis (with sufficient image quality at baseline and post-TAVI). BCVA = best corrected visual acuity. FAZ = foveal avascular zone. VD = vessel density. VLD = vessel length density. S = superficial plexus. D = deep plexus. 3 = macula 3 × 3 mm scan size, 6 = macula 6 × 6 mm scan size. i = papillary inner ring scan. o = outer papillary ring scan.

Quantitative analysis was performed on 42 eyes of the 28 patients; 38 eyes had sufficient quality macular scans for analysis (SCP and DCP and FAZ measurement), 26 eyes had sufficient quality peripapillary scans for the SCP and 24 eyes had sufficient quality peripapillary scans for the DCP. None of the measurement showed any statistically significant changes in postoperative values. Subtracting post-operative values from pre-operative values resulted in change scores oscillating around zero (the only exception being vessel density of the DCP in the outer ring around the disc), see Figure 3. Increase in left ventricular ejection fraction (LVEF) is not significantly associated with changes in OCTA measurements or BCVA (neither increase nor decrease).

### 3.4. Heat Maps

**Figure 4:**
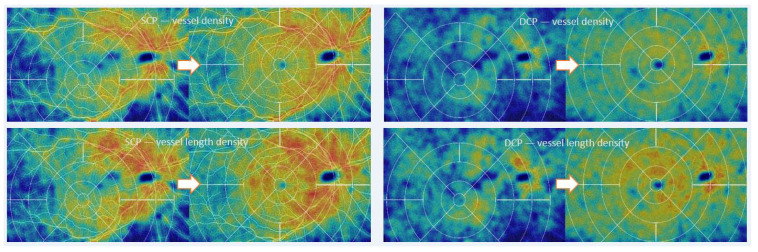
Heatmaps of 9 × 15 mm scans. Exemplary case where flow generally appears to be improved after TAVI. These images are gained by applying a data visualization technique using color hues to indicate flow intensities on standard OCTA scans. This algorithm is still under further development. The images do not represent relative blood flow speed.

Using heat maps based on an early algorithm provided by Zeiss, which is still under development, an improvement of perfusion in 9 × 15 mm scans was detected in four out of eight patients (six out of twelve eyes) and a decrease in others (Figure 4). The results are coherent between vessel density and vessel length density. Heat maps are pleasing to the eye and facilitate pattern recognition for the reader. However, clinical significance is thus far unclear, and findings need to be confirmed in a larger series.

## 4. Discussion

In our cohort, we found an incidence of 28.6% new capillary dropout lesions in OCTA scans after TAVI. The percentage of retinal embolic events might be underestimated, as the study populations was small and 13 out of 41 patients were excluded from analysis, therefore giving way for possible bias. Conducting this study as per the protocol presented with many difficulties. The general population undergoing TAVI is elderly and frail, making even non-invasive retinal imaging technique challenging. Our imaging protocol takes fifteen minutes in a healthy proband without repeating any images. In our cohort, imaging took around thirty to forty minutes including breaks in case of fatigue. OCTA is prone to artifacts in these patients, especially when acquiring the extended peripheral scans [18]. Repeatability is also a concern, as the imaging protocol was only run once with giving leeway for the imaging staff to reattempt in the case of major artifacts, limiting the quantitative analysis [19]. Several patients could not comply with the follow-up exam as they needed intensive medical attention for a TAVI related complication. The only paper in the literature to evaluate retinal emboli/events after TAVI (to our knowledge) is a series of 20 patients examined with direct and indirect biomicroscopy and fundus photography [20]. At 48 h after TAVI, they found a new cotton wool spot in one patient and at one month after TAVI, a new cotton wool spot in another patient, and a visible embolus in a third patient, resulting in an overall much lower incidence of retinal embolic events of 15%. This indicates that OCTA seems to be more sensitive and specific than biomicroscopy and/or fundus photography alone. This said, we think that this is a significant number of retinal incidences, all of which have been clinically silent and with so far unknown effects on retinal and visual function in the future. We are not aware of any publication investigating this issue at present.

Nearly all the patients (88.9%) who had MRI showed silent ischemic brain lesions after TAVI, above the 77.5% average of a recent meta-analysis [7]. As mentioned previously, only nine patients underwent baseline and post-TAVI MRI, limiting evaluation of this secondary end point. In 25% of those with ischemic brain lesions, retinal capillary dropout lesions on OCTA could be detected. Improvement in imaging technology with higher resolution of the periphery seems to be necessary to use OCTA as a surrogate marker for the burden of ischemic brain lesions in clinical daily practice. Due to the small number, correlation of silent ischemic brain lesions with capillary dropout lesions in OCT does not seem sensible in this cohort.

OCTA gives way to extensive possibilities for quantitative analysis of the central macular region. We applied commonly used parameters suitable for capturing and visualization of dropout of retinal microvascular perfusion [21]. The measurements themselves could not detect the capillary dropout lesions, assumingly too small to have a significant effect and in many cases outside of the scans used for the quantitative analysis. The overall change scores around zero might represent an effective ocular blood flow regulation in our cohort (none had more than mild diabetic and/or hypertensive retinopathy) [22]. It suggests that ocular blood flow regulation can compensate for the range of LVEF measured in this study in the central macular area. This is supported by the findings of a recent study not finding any association between vessel density on OCTA in patients with myocardial infarction, regardless of their LVEF and other hemodynamic variables [23].

Limitations of this study include logistical difficulties due to elderly and comorbid patients, which are at risk of life-threatening procedure related complications and might be unstable pre- and or post-TAVI, requiring special attention when scheduling study visits and transport. Further important limitations are technical difficulties with image acquisition, resulting in impossibility to acquire certain images due to long and strenuous acquisitions times in some cases and other images being excluded from analysis due to artifacts.

Given the fact that OCTA is a non-invasive relatively simple and cost-effective examination, it may provide a useful tool for evaluation of the risk of retinal emboli with new cardiac prostheses and embolic protection devices in the future. In addition, algorithms encoding relative blood flow speed in the macular region would allow for an additional outcome parameter for the TAVI procedure.

## Figures and Tables

**Table 1:** Patient demographics. Age-related macular degeneration was mild or intermediate, no patient had clinically significant diabetic macular edema nor proliferative diabetic retinopathy.

Baseline Characteristics	
Male gender	23 (82.1%)
Arterial hypertension	24 (85.7%)
Arterial fibrilation	2 (7.1%)
Diabetes	7 (25%)
History of strokes	2 (7.1%)
Ophthalmic baseline characteristics	
Cataract (Mild And Moderate)	12 (42.9%)
Age-Related Macular Degeneration	5 (17.9%)
Pseudophakic	8 (28.6%)
Glaucoma	2 (7.1%)
Diabetic Retinopathy	2 (7.1%)
Procedure related baseline characteristics	
Type of aortic stenosis	
- Severe high gradient aortic stenosis	15 (53.6%)
- Low-flow low-gradient aortic stenosis	8 (28.6%)
- Aortic insufficiency	3 (10.7%)
- Moderate aortic stenosis	2 (7.1%)
Valve in valve operation	2 (7.1%)

## Data Availability

The data presented in this study are available on request from the corresponding author. The data (original imaging) are not publicly available due to privacy issues.
